# High Andean Steppes of Southern Chile Contain Little-Explored *Peltigera* Lichen Symbionts

**DOI:** 10.3390/jof9030372

**Published:** 2023-03-18

**Authors:** Karla Veas-Mattheos, Katerin Almendras, Matías Pezoa, Cecilia Muster, Julieta Orlando

**Affiliations:** 1 Laboratory of Microbial Ecology, Department of Ecological Sciences, Faculty of Sciences, Universidad de Chile, Santiago 7800003, Chile; 2 Millennium Institute Biodiversity of Antarctic and Subantarctic Ecosystems (BASE), Santiago 7800003, Chile

**Keywords:** Andean steppes, Chile, cyanobiont, lichen, mycobiont, *Nostoc*, *Peltigera*, phylogeny

## Abstract

*Peltigera* lichens can colonize extreme habitats, such as high-elevation ecosystems, but their biodiversity is still largely unknown in these environments, especially in the southern hemi- sphere. We assessed the genetic diversity of mycobionts and cyanobionts of 60 *Peltigera* lichens collected in three high Andean steppes of southern Chile using LSU, *β-tubulin*, COR3 and ITS loci for mycobionts, and SSU and *rbcLX* loci for cyanobionts. We obtained 240 sequences for the different mycobiont markers and 118 for the cyanobiont markers, including the first report of *β-tubulin* sequences of *P. patagonica* through modifying a previously designed primer. Phylogenetic analyses, ITS scrutiny and variability of haplotypes were used to compare the sequences with those previously reported. We found seven mycobiont species and eleven cyanobiont haplotypes, including considerable novel symbionts. This was reflected by ~30% of mycobionts and ~20% of cyanobionts haplotypes that yielded less than 99% BLASTn sequence identity, 15 new sequences of the ITS1-HR, and a putative new *Peltigera* species associated with 3 *Nostoc* haplotypes not previously reported. Our results suggest that high Andean steppe ecosystems are habitats of unknown or little-explored lichen species and thus valuable environments to enhance our understanding of global *Peltigera* biodiversity.

## 1. Introduction

The southern Andean steppes are high-elevation vegetation zones found from latitude 27° S to 56° S, in the South American Andes of Argentina and Chile [1]. Due to a wide range of climatic conditions and different altitudes of the treelines throughout the Andes, the southern Andean steppes present potential for a high diversity of ecological niches, which can be colonized by organisms adapted to extreme conditions. Besides, high mountain peaks with exceptionally harsh climates can act as ecological islands, and the consequent phylogeographical isolation could lead to localized speciation events [2]. Therefore, these high-altitude ecosystems are a unique biodiversity reserve and are recognized centers of endemism [3,4].

Lichens are one of the most widespread and successful types of symbiosis and an important part of the cryptogamic biota in high-altitude environments [4,5] since they have a high tolerance for extreme environmental conditions [6]. These complex networks of microorganisms play an essential role in carbon, nitrogen, and phosphorus cycling [7,8,9,10]. They are formed by the interaction of a fungus (denominated mycobiont), an extracellular arrangement of one or more photosynthetic organisms (denominated photobiont/s) and a wide variety of other microorganisms, which together form the lichen thallus [11]. The mycobiont and photobiont are considered the main symbionts since they structure the thallus and are the most widely studied [12].

*Peltigera* is a genus of muscicolous and terricolous foliose lichens distributed worldwide that colonizes a wide range of environments, including high-altitude and high-latitude sites [13,14]. These lichen-forming fungi associate with cyanobacteria from the *Nostoc* genus and, in some cases, also with a green alga from the *Coccomyxa* genus [13]. In southern Chile, only bipartite representatives associated with *Nostoc* have been found [15,16]. This genus presents several cryptic species; thus, molecular analyses are necessary for the correct identification of these lichens [15,16]. Recent studies based on multi-locus phylogenies have revealed a largely underestimated level of biodiversity, with more than 150 *Peltigera* species distributed into eight sections, of which the *Peltigera* section includes the largest number of described species worldwide [13,14].

In the last few years, studies of *Peltigera* diversity based on molecular data in South America have increased, which has allowed the enriching of the databases necessary for evolutionary studies and the description of unknown or little-explored species [13,14]. Additionally, diverse studies of *Peltigera* lichen have been carried out in different environments of Chile [14,15,16,17,18,19,20]; however, the southern high Andean environments remain largely unexplored in this regard.

In this study, we focus on analyzing the genetic diversity of *Peltigera* mycobionts and cyanobionts growing in three little-explored high Andean steppes of southern Chile, along with evaluating the usefulness of the molecular markers commonly employed in the studies of these symbionts. We hypothesize that due to the hostile conditions and the scarcity of studies on biodiversity in these environments, the high Andean steppes are ecosystems with a high potential for detecting novel *Peltigera* symbionts.

To determine the genetic diversity, a molecular identification approach was used through sequencing and phylogenetic analyses of a series of markers for both symbionts, including classical markers (LSU and ITS for the mycobiont and SSU for the cyanobiont) [16,20,21,22] and novel markers (COR3 [23] for the mycobiont) as well as others little assessed in the southern hemisphere (*β-tubulin* for the mycobiont and *rbcLX* for the cyanobiont) [13].

This approach allowed us to find novel sequences for already known mycobionts and cyanobionts and a new putative species of the *Peltigera* genus associated with *Nostoc* haplotypes not previously described. Altogether these results contribute to increasing the knowledge of *Peltigera* species diversity, providing molecular data of specimens located in underexplored environments.

## 2. Materials and Methods

### 2.1. Study Sites and Sampling

Fragments (~15 cm^2^) of 60 *Peltigera* thalli in total were collected from three high An-dean steppes located in (i) Cerro Cinchao in the Coyhaique National Reserve (Aysén Region, Chile; hereafter referred to as Coyhaique), (ii) Cerro Pietrogrande in the Karukinka Natural Park (Tierra del Fuego Island, Chile; hereafter referred to as Karukinka), and (iii) Cerro Bandera close to Puerto Williams (Navarino Island, Chile; hereafter referred to as Navarino).

**Figure 1:**
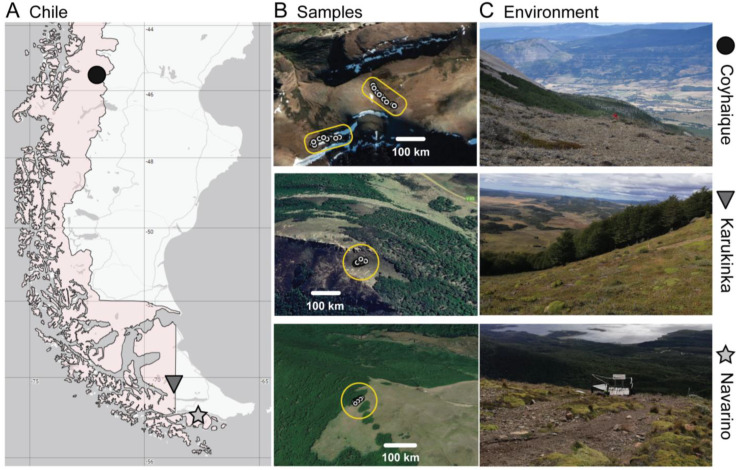
Study sites and lichen samples. (**A**) Geographic location of the three sampling sites. (**B**) Sample collection transects at each site (collection area in yellow). (**C**) Panoramic views of the sampling sites.

Coyhaique is the northernmost site, and it is located at 1250 m.a.s.l. in the Aysén region, while Karukinka and Navarino are located at ~500 m.a.s.l. in the Magallanes region. At each site, 20 lichen samples were collected at least 1 m from the next closest thallus to minimize resampling of the same genetic individual. In Coyhaique, samples were collected from two ~110 m long transects, while in Karukinka and Navarino, samples were collected from a ~40 m long transect (Figure 1, Table S1). The collected portions of lichen thalli were superficially cleaned with a sterile brush and spatula, and ~0,025 g of each thallus were mechanically fractionated with a homogenizer FastPrep^®^-24 (MP Biomedicals, OH, USA) for 30 s at 6 m/s. DNA was extracted by the CTAB method following the protocol from Gardes and Bruns [24] with modifications, specifically an increase in the cold incubation time to 2 h and 16 h for the first and second incubation, respectively, and an increase in the time of all the centrifugations to 15 min. The extracted DNA was stored at −20 °C until analysis.

### 2.2. Molecular Data

For the mycobiont identification, four molecular markers were targeted. We amplified: (i) ~1.1 kb of the large subunit rDNA gene (LSU) using LIC24R [21] and LR7 [25] primers; (ii) ~0.6 kb of the *β-tubulin* gene using the forward primers T1 [26], bt_34F [27] or bt_34F_mod (5′-CACGCGTCTATTCCATATCC-3′) and the reverse primer BT2B [28]; (iii) ~0.7 kb of a collinear orthologous region 3 (COR3) using COR-3F-A and COR-3R-B primers [23]; and (iv) ~0.6 kb of the internal transcribed spacer (ITS) using the forward primer ITS1F [24] and the reverse primer ITS4 [29]. Due to the absence of *β-tubulin* marker amplification for some samples, the bt_34F primer was modified using *β-tubulin* sequences obtained from the dataset available in the Tree-Based Alignment Selector toolkit v2.2 [30] as a reference, and a new primer (bt_34F_mod) which preserves the 3′ region of the bt_34F primer was designed. For the cyanobiont identification, we amplified two molecular markers: (i) ~1.0 kb of the small subunit rDNA gene (SSU) using PCR1 and PCR18 primers [31] and (ii) ~1.0 kb of the *rbcLX* region using CW and CX primers [32].

All amplifications were carried out in a 25 µL reaction volume containing GoTaq^®^ Green Master Mix (Promega, WI, USA), 0.2 mM of each primer, and 10 ng of genomic DNA in a Maxygene thermocycler (Axygen, CA, USA). In cases of absence of amplification or non-specific amplification, BSA was incorporated at a maximum concentration of 1 µg/µL. PCR conditions for fungal LSU and bacterial SSU follow Zúñiga et al. [16] and the literature cited therein. The conditions to amplify the fungal *β-tubulin* marker differed according to the primers used. For bt_34F/BT2B primers, the conditions were: initial denaturation of 5 min at 95 °C; followed by 35 cycles of 45 s at 95 °C, 1.5 min at 52 °C and 1.5 min at 2 °C; with a final extension of 10 min at 72 °C. For bt_34F_mod/BT2B and T1/BT2B primers, the conditions were: initial denaturation of 4 min at 94 °C; 35 cycles of 1 min at 94 °C, 1.5 min at 60 °C and 1.5 min at 72 °C; followed by a final extension of 10 min at 72 °C. The fungal COR3 region was amplified using the following conditions: initial denaturation of 2 min at 94 °C; 30 cycles of 1 min at 94 °C, 1 min at 49.6 °C and 1.5 min at 72 °C; followed by a final extension of 10 min at 72 °C. To amplify the fungal ITS region, the following conditions were used: initial denaturation of 1 min at 94 °C; 30 cycles of 1 min at 94 °C, 1 min at 55 °C and 2 min at 72 °C; followed by a final extension of 7 min at 72 °C. Finally, to amplify the bacterial *rbcLX* region, the following conditions were used: initial denaturation of 4 min at 94 °C; 30 cycles of 1 min at 94 °C, 1 min at 63 °C and 2 min at 72 °C; followed by a final extension of 7 min at 72 °C.

The quality and size of the amplicons were visualized using 2% (*w*/*v*) agarose gels in TAE 1x buffer (40 mM Tris-acetate, 1mM EDTA [pH 8.0]) stained with GelRed^TM^ (Biotium, CA, USA), and PCR amplicons were stored at 4 °C. In cases of non-specific amplifications, the band of interest was cut from the agarose gel and purified using the Wizard^®^ SV Gel and PCR Clean-Up System kit (Promega, WI, USA). All amplicons were sequenced using the forward primers by a sequencing service (Macrogen, Seoul, Republic of Korea) in a Genetic Analyzer 3730XL (Applied Biosystems, CA, USA). Sequences that present a chromatogram with uncertain regions due to polymerase slippage at the poly A or poly T region were re-sequenced using reverse primers, and consensus sequences were constructed to obtain the full sequence. DNA sequences were visually checked and manually edited on the SnapGene software (Insightful Science; http://www.snapgene.com, accessed on 28 September 2022) and Mega X software [33]. For each marker, the sequences were aligned using MAFFT version 7 (https://mafft.cbrc.jp, accessed on 28 September 2022) [34] and were grouped in haplotypes by a visual check using Mega X [33] (Table S1). The sequences obtained were deposited in the GenBank database [35] (Table S1), and one representative of each haplotype was compared with sequences in the NCBI database using BLASTn [36] (Table S2).

### 2.3. Mycobiont Identification

#### 2.3.1. Phylogenetic Analyses

To determine to which *Peltigera* clades our samples belong, the phylogenetic placement of the LSU haplotypes was carried out with the Tree-Based Alignment Selector toolkit (T-BAS) [30] using a *Peltigera* reference tree at the genus level. The Evolutionary Placement Algorithm (EPA) [37] was applied with the GTR substitution model [38] and gamma distribution parameter (GTRGAMMA).

Then, multi-locus phylogenetic analyses were carried out. First, a reference database was built with sequences of *β-tubulin*, COR3, and ITS markers based on published data of currently validated species sequences from clades identified in the previous analysis: clade 1 (*P. retifoveata*), clade 2 (*P. frigida*/*P. patagonica*), clade 5 (*P. ponojensis*/*P. monticola*), clade 6 (*P. rufescens*), and clade 9 (*P. canina*) [13]. At least one representative of each species was selected, except for *P. frimbiata*, for which only ITS was available, and it was discarded from the reference set after confirming that it had no similarity with the sequences of this study. In addition, *P. antarctica* individuals were named *Peltigera* sp. 23 since a DNA sequence from the type specimen of *P. antarctica* allowed Miadlikowska and Magain (personal communication) [39] to confirm that it was not what was called *P. antarctica* in Magain et al. [13]. Sequences retrieved from BLASTn searchers were also incorporated. Thus, the reference database comprised 402 sequences belonging to 139 individuals (Table S3) [13,27,37,40,41].

For each marker, the reference sequences and those from our samples were aligned by MAFFT version 7 [34]. The alignment was manually edited using MEGAX [33], and ambiguously aligned regions were delimited *sensu* Lutzoni et al. [42] and were excluded from phylogenetic analyses. The markers were delimited, and the following data subsets were separately analyzed: *β-tubulin*, ITS1, 5.8S, ITS2, and COR3. MrModelTest2 [43] was used to determine the optimal model.

The sequences of the three markers were concatenated using MEGAX, and the multi-locus sequences were grouped in operational taxonomic units (OTUs; 100% nucleotide identity of edited and concatenated sequences). Maximum likelihood (ML) and Bayesian (BI) analyses were carried out using *P. retifoveata* sequences as an outgroup. Searches for the optimal tree and bootstrap analysis (1000 replicates, GTRGAMMA substitution model) were performed using RAxML v.8.2.12 [44] available on the CIPRES portal [45]. BI analysis was carried out for 20 million generations, sampling every 2000th generation with MrBayes v.3.2.7 [46] as implemented on the CIPRES portal [45]. The phylogenetic trees were edited in the Interactive Tree of Life platform (iTOL) (https://itol.embl.de/, accessed on 31 October 2022) [47] and Adobe Illustrator CC 2019 (23.0.3) (Adobe Inc., San Jose, CA, USA).

#### 2.3.2. ITS1 Hypervariable Region Analyses

To complement the identification of mycobionts, we analyzed the ITS1 spacer of the hypervariable region (ITS1-HR) of the samples, comparing them with those of the reference sequences [13]. For this, the ITS sequences of the references and samples were grouped by species and re-aligned using MUSCLE [48] in MEGAX [33]. The ITS1-HR was determined, and the rest of the sequence was excluded. In cases where the same ITS1-HR was found in more than one species, the entire ITS sequence was analyzed.

#### 2.3.3. Haplotype Diversity

To explore the genetic diversity of the mycobionts and their distribution in the different sampling sites, haplotype networks for each *Peltigera* species identified and for each marker (LSU, *β-tubulin*, COR3 and ITS) were generated using the program Network version 10.2 [49]. Each data set was divided into seven subgroups based on sequence similarity (*P. aubertii*, *Peltigera* sp. 23, *Peltigera* sp. 24, *P. frigida*, *P.* “*fuscopraetextata*”, *P. patagonica* and *P. rufescens*) so there were no ambiguous positions in the alignment. The haplotypes were connected using the median-joining method, and gaps were considered a fifth-character state. The networks were edited using Adobe Illustrator (Adobe Inc., San Jose, CA, USA).

### 2.4. Cyanobiont Identification

The *Nostoc* SSU sequences were compared with those of other studies of *Peltigera* lichens from Chile [8,16,20,28,50,51]. We performed phylogenetic analyses on the *rbcLX* dataset. For this, a reference dataset was built with *rbcLX* sequences of symbiotic and free-living *Nostoc* species from Magain et al. [13] and other sequences available in GenBank [35] (Table S3). The reference sequences and one sequence representative of each haplotype of this study were aligned using MAFFT version 7 [34]. The alignment was manually edited using MEGAX [33], and ambiguously aligned regions were removed from phylogenetic analyses. An ML search for the optimal tree and a bootstrap analysis of the dataset were performed using RaxML version v.8.2.12 [44] as implemented on the CIPRES portal. MrModelTest2 [43] was used to determine the optimal model, and BI analysis was carried out for 20 million generations, sampling every 2000th generation with MrBayes v.3.2.7 [52] as implemented on the CIPRES portal [45].

## 3. Results

### 3.1. Mycobiont Identification

The mycobionts of 60 *Peltigera* lichens collected from three different high Andean steppes in southern Chile were identified by targeting four molecular markers: LSU, *β-tubulin*, COR3, and ITS. Some samples did not show amplification of *β-tubulin*, so a new forward primer, bt_34F_mod, was generated. This primer allowed us to obtain the missing amplicons (subsequently identified as eight *P. patagonica*, one *P. frigida* and one *Peltigera* sp. 23 representatives). This way, it was possible to obtain all the sequences of the four markers, resulting in a total of 240 sequences.

The sequences were grouped into haplotypes, and it was possible to identify 8 haplotypes for LSU, 11 for *β-tubulin*, 13 for COR3, and 27 for ITS (Table S1). A BLASTn analysis confirmed that all haplotypes corresponded to the *Peltigera* genus (Table S2). Nevertheless, 2 *β-tubulin* haplotypes (TUB_03 and 04), 3 COR3 haplotypes (COR_09, 10 and 11) and 13 ITS haplotypes (ITS_03 to 10 and ITS_19 to 23) yielded sequence identity values less than 99% (Table S2).

Then, the phylogenetic placement of the eight LSU haplotypes was carried out with T-BAS [30]. This analysis allowed us to classify the sequences into four clades, clade 2 (*P. frigida*/*P. patagonica*), clade 5 (*P. ponojensis*/*P. monticola*), clade 6 (*P. rufescens*), and clade 9 (*P. canina*) (Figure S2). Next, a multi-locus phylogenetic analysis was performed. For this, a reference set of sequences of *β-tubulin*, COR3, and ITS markers of *Peltigera* species belonging to these four clades was constructed. Of the total specimens in the reference set, 89.05% were represented by sequences of the three markers. The remaining 10.95% were represented only by two markers, corresponding to representatives of *Peltigera* sp. 16, *Peltigera* sp. 22, *P. retifoveata*, and *P. patagonica*. *β-tubulin* sequences were the most absent of the set (8.76%), followed by COR3 (1.46%) and, lastly, ITS (0.73%) (Table S3). Finally, the sequences of the references and samples were concatenated and grouped in OTUs (100% nucleotide identity of edited and concatenated sequences), and 153 unique multi-locus sequences were obtained, including 23 OTUs of the samples to be identified (Tables S1 and S3).

**Figure 2:**
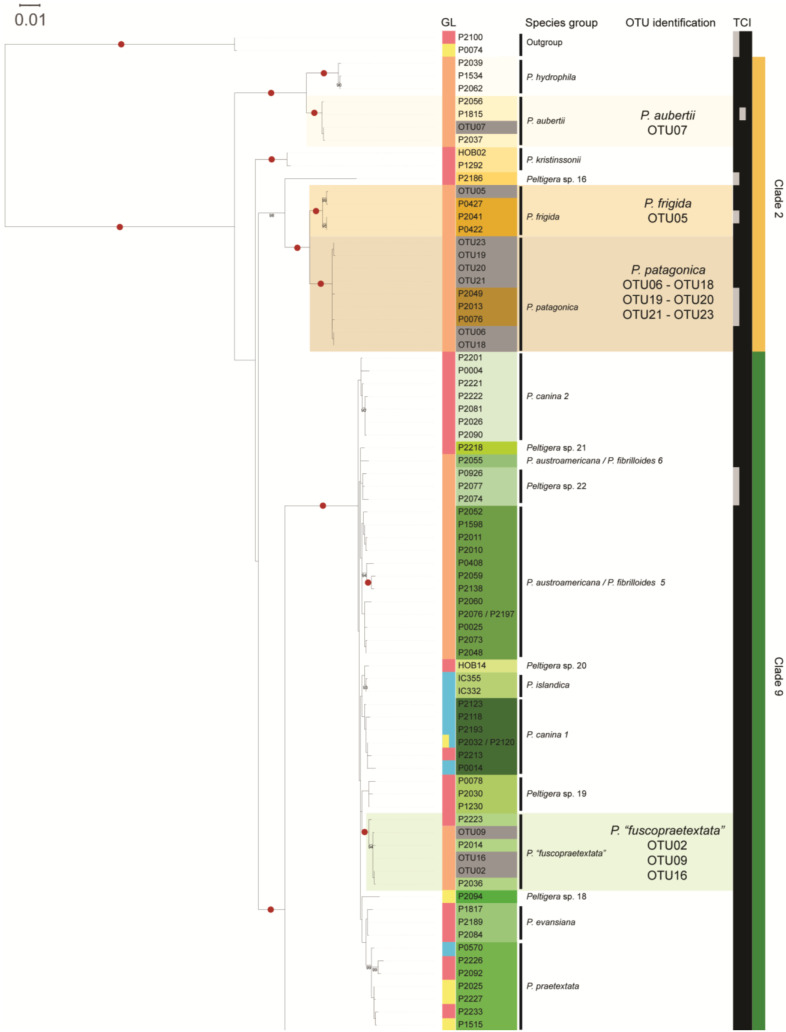

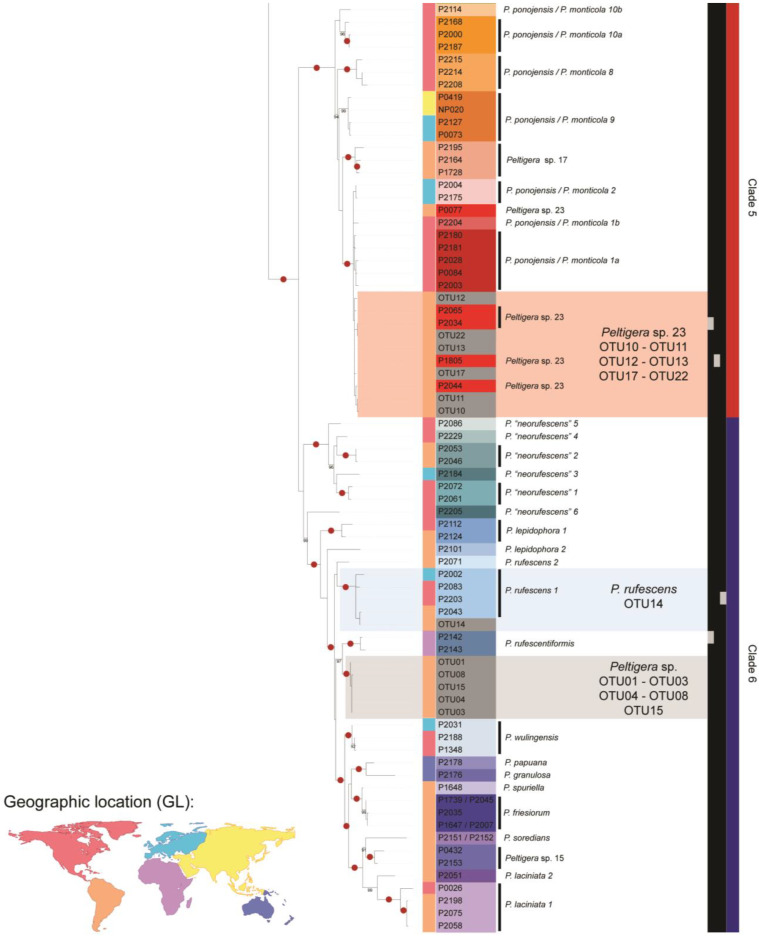
*Peltigera* best ML tree resulting from a RaxML analysis of a concatenated dataset of three loci representing 128 multi-locus reference sequences, 23 sample sequences and 2 outgroup sequences from section *Retifoveatae*. The label of each reference sequence is colored according to the species delimited by Magain et al. [13] or Miadlikowska and Magain (personal communication) [39], which are indicated next to the tree clusters. OTUs generated in this study are indicated in gray. Color boxes represent the geographical location (GL) from where the samples were collected, and black boxes represent the sequenced loci in the following order: *β-tubulin* (T), COR3 (C) and ITS (I). The clades in the tree are represented by a color bar numbered according to Magain et al. [13]. Bootstrap support ≥ 90% is indicated with numbers, and support of 100% is indicated with red circles.

ML and BI phylogenetic analyses were performed on this set of sequences and showed similar tree topologies. The sequences were grouped into four clades: three highly bootstrap-supported monophyletic clades within the genus *Peltigera*—clade 5 (*P. ponojensis*/*P. monticola*), clade 6 (*P. rufescens*), and clade 9 (*P. canina*)—and clade 2 (*P. frigida*/*P. patagonica*), which was subdivided into three highly supported subclades—clade 2a (*P. hydrophila*/*P. aubertii*), clade 2b (*Peltigera* sp. 16/*P. frigida*/*P. patagonica*), and clade 2c (*P. kristinssonii*) (Figure 2). The 23 OTUs to be identified were closely associated with seven groups, five of them highly supported in monophyletic groups with a single species: *P. aubertii* (OTU07), *P. frigida* (OTU05), *P. patagonica* (OTU06, 18, 19, 20, 21, and 23), *P.* “*fuscopraetextata*” (OTU02, 09 and 16), and *P. rufescens* (OTU14). The sixth group (OTU10, 11, 12, 13, 17, and 22) was found to be highly supported in a monophyletic clade containing species of *Peltigera* sp. 23 and putative species of *P. ponojensis*/*P. monticola* (1a, 1b, and 9), but the presence of an internal node would indicate that the OTUs were closer to *Peltigera* sp. 23 specimens, although with low support. The seventh group (OTU 01, 03, 04, 08, and 15) was found in a highly supported monophyletic clade with no other species but which formed a sister group with *P. rufescentiformis* (Figure 2).

**Figure 3:**
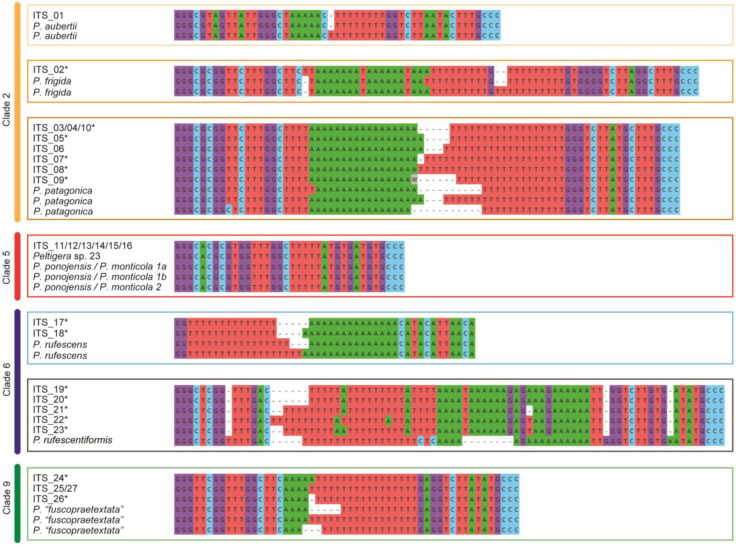
ITS1-HR sequences of the samples from this study and the closest reference sequences grouped by *Peltigera* clades, according to Magain et al. [13]. Asterisks indicate novel sequences of ITS1-HR.

Additionally, the sequences of the ITS1-HR of the samples were compared with reference sequences to complement the identification of mycobionts. The OTUs associated with *P. aubertii*, *P. frigida* and *Peltigera* sp. 23 presented a single ITS1-HR sequence, while OTUs associated with *P. patagonica*, *P. rufescens*, *P.* “*fuscopraetextata*” and *Peltigera* sp. 24 presents two to six different sequences of the region (Figure 3). A total of 19 unique sequences of the ITS1-HR were found for the 27 ITS haplotypes, among which 15 were not previously reported (Figure 3).

The ITS1-HR sequence of the OTUs previously associated with *P. aubertii*, *P. frigida*, *P. patagonica*, *P. rufescens* and *P.* “*fuscopraetextata*” were similar or identical to some from reference species, supporting the identification results previously obtained. However, the OTUs associated with *Peltigera* sp. 23 were identical to more than one species. Therefore, this analysis did not allow us to confirm the identity of this group of OTUs, and the complete ITS region was analyzed. In this analysis, three regions presented differences between the sequences of *Peltigera* sp. 23 and *P. ponojensis*/*P. monticola*, allowing the assignment of ITS haplotypes to *Peltigera* sp. 23 (Figure S2). Last, the ITS1-HR sequence of the OTUs not associated with any species (OTU01, 03, 04, 08, and 15) differed from the one from *P. rufescentiformis*, the closest species according to the phylogenetic analysis, reinforcing the idea that this group could correspond to a previously unidentified species, which was denominated as *Peltigera* sp. 24 (Figure 3).

Through the complementation of phylogenetic analyses and review of the ITS1-HR, the results suggest the presence of seven species for the 60 *Peltigera* lichen collected from the three sites. Of these, 6 corresponded to previously recognized or putative species: *P. aubertii* (*n* = 1), *P. frigida* (*n* = 1), *P. patagonica* (*n* = 16), *Peltigera* sp. 23 (*n* = 10), *P. rufescens* (*n* = 2), *P.* “*fuscopraetextata*” (*n* = 20), and one corresponding to a putative new species belonging to the clade *P. rufescens*, which was named *Peltigera* sp. 24 (*n* = 10).

**Figure 4:**
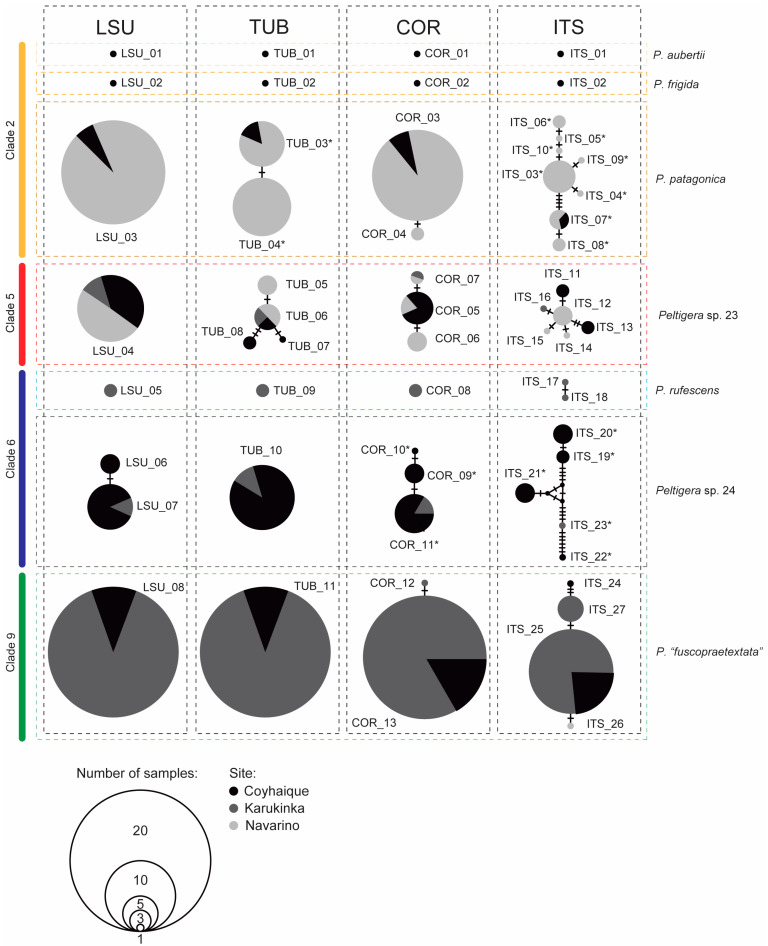
Networks of *Peltigera* mycobiont haplotypes based on four fungal markers: LSU, *β-tubulin* (TUB), COR3 (COR), and ITS. The graphical representation of the number of samples and colors of the sites are shown in the lower left corner. Asterisks indicate sequences of the markers yielding percentage identity values less than 99%, according to BLASTn (Table S2).

Finally, the value of molecular markers to determine the genetic diversity of the mycobionts within each *Peltigera* species was assessed using networks of haplotypes (Figure 4). For the LSU marker, we found only one haplotype per species, even in species found in two or three sites. The exception was *Peltigera* sp. 24, which presented two different LSU haplotypes that varied in a single nucleotide: LSU_06 and LSU_07 were found in Coyhaique, and only LSU_07 was found in Karukinka. For the *β-tubulin* marker, a single haplotype was found for most species, except for *P. patagonica* and *Peltigera* sp. 23, which presented two and four different *β-tubulin* haplotypes, respectively. In the case of the COR3 marker, *P. aubertii*, *P. frigida*, and *P. rufescens* exhibited only one haplotype, *P. patagonica* and *P.* “*fuscopraetextata*” presented two COR3 haplotypes, and *Peltigera* sp. 23 and *Peltigera* sp. 24 presented three different haplotypes each. Finally, the ITS marker yielded the greatest richness of haplotypes for all species, reaching eight different ITS haplotypes in the case of *P. patagonica.* For all the markers, at least one haplotype only present in one of the study sites was found, with Coyhaique and Navarino being the sites harboring the highest number of exclusive haplotypes (13 and 14, respectively).

### 3.2. Cyanobiont Identification

The cyanobiont of the 60 *Peltigera* lichens were analyzed by targeting the SSU and *rbcLX* molecular markers, obtaining 58 and 60 sequences, respectively. The sequences were grouped in haplotypes, and it was possible to identify 7 haplotypes for the SSU marker and 11 for the *rbcLX* marker. A BLASTn analysis on the sequences of each haplotype confirmed that all correspond to *Nostoc*, except the SSU_06 haplotype, which was associated with an uncultured bacterium (Table S2). One SSU haplotype (SSU_01) and three *rbcLX* haplotypes (RBC_01, 02 and 03) yielded sequence identity values of less than 99% (Table S2).

**Figure 5:**
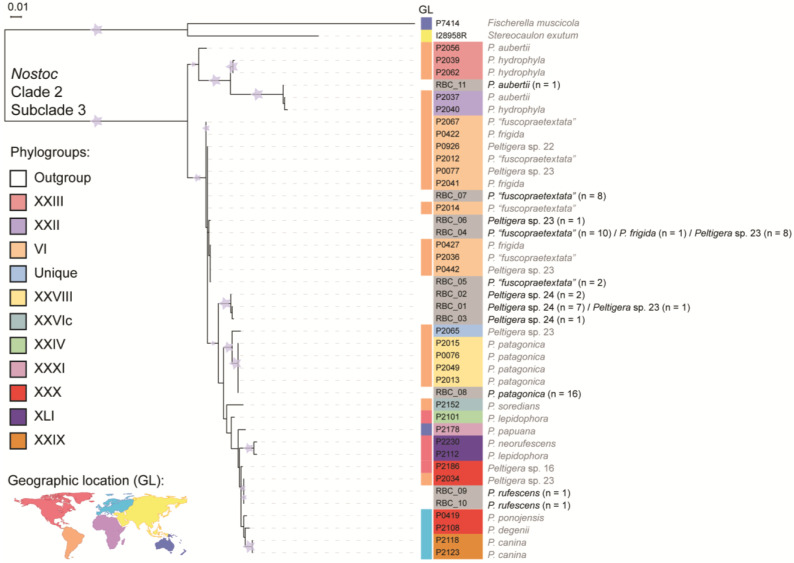
Phylogenetic analysis of *Nostoc* cyanobionts. The best ML tree resulting from a RaxML analysis of the *rbcLX* locus from 31 reference sequences, 11 sample sequences and 2 outgroup sequences from *Fischerella muscicola* and *Nostoc* from *Stereocaulon exutum* is shown (Tables S1 and S3). Each sample is colored according to the phylogroups I–VI delimited by O’Brien et al. [27] and phylogroups VII–XLVIII delimited by Magain et al. [13]. The *Peltigera* mycobiont of each *Nostoc* representative is next to the tree nodes. Color boxes represent the geographical localization (GL) from where the sample was collected. Branches with stars received bootstrap support ≥ 75%. The *rbcLX* haplotypes generated in this study are indicated in grey.

The *rbcLX* marker was used for ML and BI phylogenetic analyses, exhibiting both similar tree topologies. The *rbcLX* phylogeny revealed that all the cyanobionts of this study belong to *Nostoc* clade 2, subclade 3 (Figure 5). Some haplotypes grouped with reference sequences of phylogroups VI (RBC_04, 05, 06, and 07), XXX (RBC_09, and 10), XXVIII (RBC_08), and XXII (RBC_11). *Nostoc* representatives of these phylogroups were associated with various *Peltigera* species, except for phylogroup XXVIII, which was exclusively associated with *P. patagonica*. The RBC_01, RBC_02, and RBC_03 haplotypes clustered into a highly supported monophyletic clade, which was not closely associated with any sequence in the dataset, suggesting a new group of *Nostoc* cyanobionts (denominated NA: not assigned).

### 3.3. Peltigera Symbionts in the High Andean Steppes

**Figure 6:**
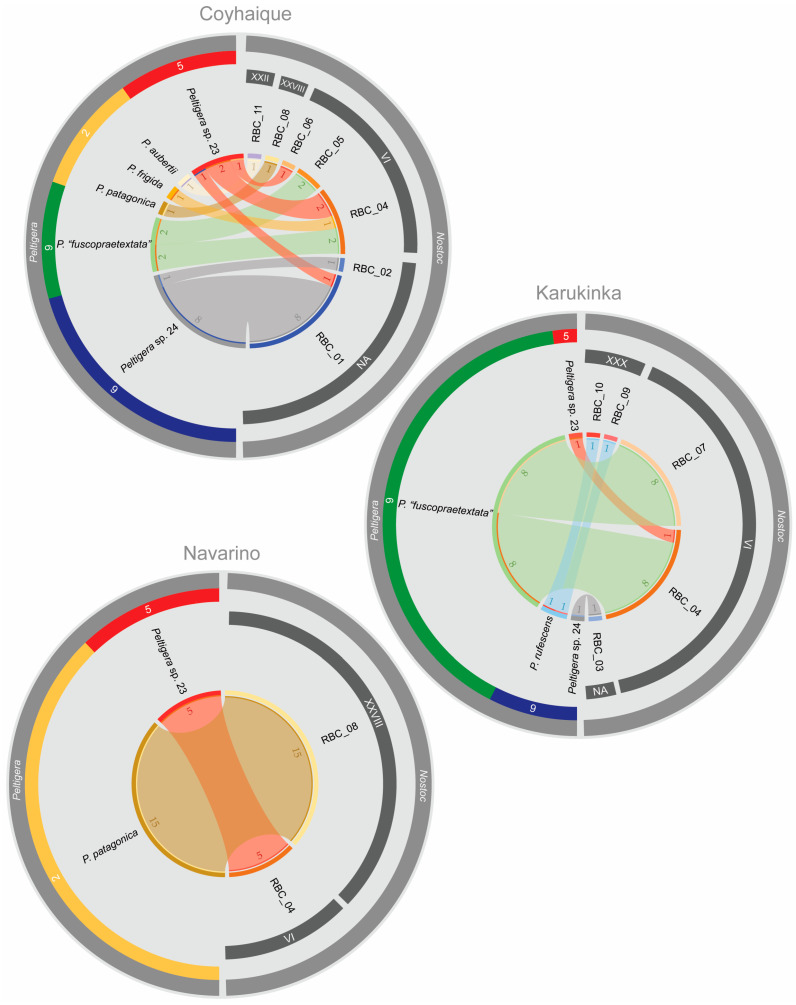
Symbiotic associations of mycobionts (*Peltigera* species) and cyanobionts (*Nostoc* haplotypes) in the three high Andean steppes plotted using Circos Table Viewer v0.63-10 [53] and edited in Adobe Illustrator. The width of the ribbons shows the abundance of the associated symbionts. The outer stripes indicate the identified symbionts (*Peltigera* or *Nostoc*), while the inner stripes indicate the *Peltigera* clades or *Nostoc* phylogroups.

The three high Andean steppes presented different mycobiont species compositions (Figure 6). Six different species were found in Coyhaique: *P. aubertii*, *P. frigida*, *P.* “*fuscopraetextata*”, *P. patagonica, Peltigera* sp. 23, and *Peltigera* sp. 24; four in Karukinka: *P.* “*fuscopraetextata*”, *P. rufescens, Peltigera* sp. 23, and *Peltigera* sp. 24; and only two in Navarino: *P. patagonica* and *Peltigera* sp. 23. A particular species dominated in each site: *Peltigera* sp. 23 in Coyhaique, *P.* “*fuscopraetextata*” in Karukinka, and *P. patagonica* in Navarino.

On the other hand, Coyhaique was also the site with the highest richness of cyanobionts, presenting seven *rbcLX* haplotypes (RBC_01, 02, 04, 05, 06, 08, and 11) corresponding to four different *Nostoc* phylogroups (VI, XXII, XXVIII, and NA) (Figure 6). In Karukinka, five *rbcLX* haplotypes were found (RBC_03, 04, 07, 09, and 10), corresponding to three different *Nostoc* phylogroups (VI, XXX, and NA). Meanwhile, in Navarino, only two *rbcLX* haplotypes were found (RBC_04 and 08), corresponding to two *Nostoc* phylogroups (VI and XXVIII). RBC_01 was the most abundant haplotype in Coyhaique, while it was RBC_04 in Karukinka, and RBC_08 in Navarino.

Finally, we analyzed the associations between mycobionts and cyanobionts (Figure 6). *P. aubertii*, *P. frigida* and *P. patagonica* were associated with a single *Nostoc* haplotype (RBC_11, 04 and 08, respectively). *P. rufescens* was associated with two *Nostoc* haplotypes (RBC_09 and 10, both belonging to the phylogroup XXX). The remaining three *Peltigera* species were found to be associated with three cyanobionts haplotypes: *P.* “*fuscopraetextata*” with RBC_04, 05, and 07 (phylogroup VI); *Peltigera* sp. 23 with RBC_04, RBC_06 (phylogroup VI), and RBC_01 (phylogroup NA); and *Peltigera* sp. 24 with RBC_01, 02, and 03 (phylogroup NA).

## 4. Discussion

Despite the considerable increase in DNA sequences available due to recent *Peltigera* phylogeny studies, the most significant sampling efforts come from the northern hemisphere; hence, those corresponding to lichens with distributions restricted to the southern hemisphere are still scarce. This was evidenced, for instance, through our BLASTn analysis, in which ~30% of the mycobiont haplotypes yielded less than 99% sequence identity. Although this search was performed in a not-exhaustively curated database, we found that values less than 99% of sequence identity coincided with (i) little-explored endemic specimens (i.e., TUB_03 and 04, associated with *P. patagonica*), (ii) specimens not identified at the species level (i.e., *Peltigera* sp. 24), (iii) a high novelty of ITS sequences, and (iv) *Nostoc* haplotypes associated with unidentified *Peltigera* species. Therefore, we consider it an extra criterion to reveal the novelty of sequences obtained from our sampling sites.

However, the most remarkable example of the limited information available for some specimens restricted to the southern hemisphere is the absence of *β-tubulin* sequences for *P. patagonica* in the databases. In our study, it was possible to obtain *β-tubulin* sequences from this species for the first time, along with some from *P. frigida* and *Peltigera* sp. 23 individuals, by generating a modification of the bt_34f primer [27]. These *Peltigera* species have a restricted distribution in the southern hemisphere, and their inclusion in phylogenies could reveal the evolutionary history of the *Peltigera* genus since it has been proposed that the most recent common ancestor of the *Peltigera* section could have inhabited the Neantarctic biogeographic region [13].

Although our study was on a small scale, the phylogeny agreed with the topology of more exhaustive phylogenetic analyses based on a large set of species worldwide [13,21,54]. Indeed, our set of sequences grouped with the least support coincided with groups of species previously difficult to resolve, such as *P. canina* and *P. ponojensis*/*P. monticola* clades [13,54,55]. The only case of a group of OTUs not grouped with other previously reported species was named *Peltigera* sp. 24. They formed a monophyletic clade sister to *P. rufescentiformis* sequences, a group of lichens until now found exclusively in Africa [17].

To complement the phylogenetic analyses, the ITS1-HR was assessed. This region varies significantly in length and can contain microsatellites with single nucleotide strings or short repeats; therefore, it is normally excluded in phylogenetic analyses [13]. The analysis of this region of our samples revealed a high number of ITS1-HRs previously not reported in the literature, and this contributed to new sequences for the databases of *Peltigera* lichens from the Southern Cone. Although the ITS1-HR constitutes a powerful marker for identifying *Peltigera* section species [13], it was not useful for *Peltigera* sp. 23 identification because its sequence is identical to that of *P. ponojensis*/*P. monticola* 1 and 2. Therefore, the analysis of a wider region of the ITS marker surrounding the ITS1-HR would be a valuable tool for the identification of this species with a restricted distribution in the southern part of the world; even more so, considering that it belongs to one of the undefined groups in the *Peltigera* phylogeny [13,54]. We expect that the increase of sequences from representatives of this clade may contribute to resolving this issue.

Last, we evaluated the potential of each marker to determine the genetic intraspecific variability. Considering the most abundant *Peltigera* species, LSU was the most conserved marker and the ITS the most variable one, which confirms the acknowledgement of the ITS region as a universal DNA barcode for fungi [56]. Although the resolution of the COR3 and *β-tubulin* markers varies according to the species analyzed, they provide useful information in detecting haplotypes exclusive to one of the studied sites, which can be especially valuable for conservation and biodiversity management plans [57,58].

On the other hand, the *Nostoc* cyanobionts cannot currently be named at the species level; however, haplotypes or genotypes can be identified using molecular markers such as *rbcLX* and SSU [59]. Because most of the studies in *Peltigera* lichens from Chile have used the SSU marker to identify the *Nostoc* cyanobionts [16,18,20,50,51], a comparison of the SSU haplotypes with those reported in the literature was made. Nevertheless, since the *rbcLX* region has been successfully used in the most recent studies to perform molecular phylogenies in *Nostoc* cyanobionts [13,23,27] and showed the highest novelty in the BLASTn analysis, we delimited the *Nostoc* haplotypes using this marker.

The choice of markers for the study of lichen symbionts should consider not only the focus of the study but also the feasibility of the sequence acquisition, the variability of molecular markers, and their availability in databases [60]. Therefore, conserved molecular markers, such as LSU for mycobionts and SSU for cyanobionts, would be useful in exploratory analyses, allowing a fast screening of a large set of samples. Conversely, more variable markers for each symbiont would contribute to a more accurate analysis of genetic diversity, allowing detailed analyses of population genetics, evolution, and phylogeography, among others.

Altogether, these markers revealed a genetic diversity of *Peltigera* mycobionts and *Nostoc* cyanobionts not previously described. A total of seven *Peltigera* species were found in the studied high Andean steppes: *P. aubertii*, *P. frigida*, *P.* “*fuscopraetextata*”, *P. patagonica*, *P. rufescens*, *Peltigera* sp. 23 and *Peltigera* sp. 24, the last two not being conclusively identified. *Peltigera* sp. 23 was named this way instead of *P. antarctica*, as proposed by Magain et al. [13], due to its correct assignment being still controversial (Miadlikowska and Magain, personal communication [39]). *Peltigera* sp. 24 formed a well-supported clade closest to *P. rufescentiformis*, then corresponding to a new putative species of the *P. rufescens* clade [13].

Among the seven found species, only *P.* “*fuscopraetextata*” and *P. rufescens* have wide distributions. *P.* “*fuscopraetextata*” has been reported as a bipolar species [13,16,61,62], which could explain its ability to colonize high mountain sites [63]. *P. rufescens* has been described as a cosmopolitan species with a preference for habitats free of tree cover and is tolerant to high UV exposure and low nutrient availability [13,15,62,64]. The rest of the *Peltigera* species found in the high Andean steppes have been reported exclusively in Chile and Argentina [13,17,19,20,62].

On the other hand, 11 *Nostoc* haplotypes were found, of which three (RBC_01, RBC_02, and RBC_03) were not closely associated with any previously reported representative and clustered into a highly supported monophyletic clade, suggesting a new group of *Nostoc* cyanobionts. These were associated with the putative new species *Peltigera* sp. 24, which was exclusively related to members of this *Nostoc* clade. While more approaches are necessary, it is likely that *Peltigera* sp. 24 would correspond to a specialist species since it was only associated with phylogenetically close cyanobiont haplotypes. These *Nostoc* haplotypes could correspond to an unreported strain because of the low percentage of identity found for their sequences in the database, the particularity of the studied environments, and the probable coevolution of mutualistic interactions [65].

This group of *Nostoc* could be adapted to high Andean steppes by presenting interesting survival strategies to withstand these harsh environments. The photobiont partner is crucial in the whole symbiosis survival in these conditions since it participates through different mechanisms to mitigate or tolerate different stressors [6,66], which could be aggravated due to climate change [67]. This and other studies [14,37,68,69,70,71,72] highlight the importance of performing studies in still-understudied areas regarding biodiversity, which can be essential for studying adaptation strategies. Considering the particularity of the climatic factors in the studied environments and their influence in shaping photobiont distributions and their association patterns [23,73,74], the genetic diversity found in this work may not be widely distributed in the whole Andean landscape but is restricted to specific patches.

Studies of *Peltigera* biogeographic patterns have suggested that climatic variables impact the species distribution more than geographic distance [13,73]. The characteristics of the studied sites, both due to their global location and altitude, coincide with the geographic and climatic ranges reported as suitable for the found *Peltigera* groups [13,15,16,18]. High mountain peaks with an exceptionally cold and harsh climate act as islands [2], and geographical isolation can promote speciation events and endemism [3,75]. Therefore, even though our study includes only three high Andean steppes, the occurrence of endemic species and the presence of a potential new species above the treeline could be related to elevation since that is known to favors isolation [76].

Our study sites are areas of high vulnerability regarding the effects of climate change [67], and therefore expanding the knowledge of their biological diversity is essential for the future application of conservation strategies in these environments. High Andean steppes are a reservoir of novel diversity for species of endemic cryptogamic biota [71,77]. Indeed, they are a refuge for an unexplored diversity of *Peltigera* lichens, including previously unreported species and novel sequences of already identified species [13,14,16]. The exploration of these environments would allow the knowledge of biological diversity in these areas to be enriched as well as contribute to global data from isolated regions.

## Data Availability

GenBank accession numbers of the newly generated sequences can be found in Table S1.

## Supplementary Materials

The following supporting information can be downloaded at: https://www.mdpi.com/article/10.3390/jof9030372/s1, Table S1. Geo-reference and identification information of the samples analyzed in this study. Table S2. BLASTn information for the sequences closest to the haplotypes of this study. Table S3. Information of the *Peltigera* and *Nostoc* reference sequences used in this study. Figure S1. Evolutionary Placement Algorithm as implemented in the Tree-Based Alignment Selector toolkit (T-BAS) using the genus *Peltigera* reference tree-based LSU sequences. Figure S2. Alignment of ITS haplotypes associated with clade 5 with the closest reference sequences according to phylogenetic analysis. Representatives of *P. antarctica* and *P. ponojensis*/*P. monticola* 1a, 1b and 2 are included.
