# Supplementary material for: High Andean Steppes of Southern Chile Contain Little-Explored Peltigera Lichen Symbionts
Source: J Fungi (Basel). 2023 Mar 18;9(3):372. doi: 10.3390/jof9030372 (PMC10058012; doi:10.3390/jof9030372)
Supplement: Supplementary file 1 [file jof-09-00372-s001.zip › Figure S2.pdf]

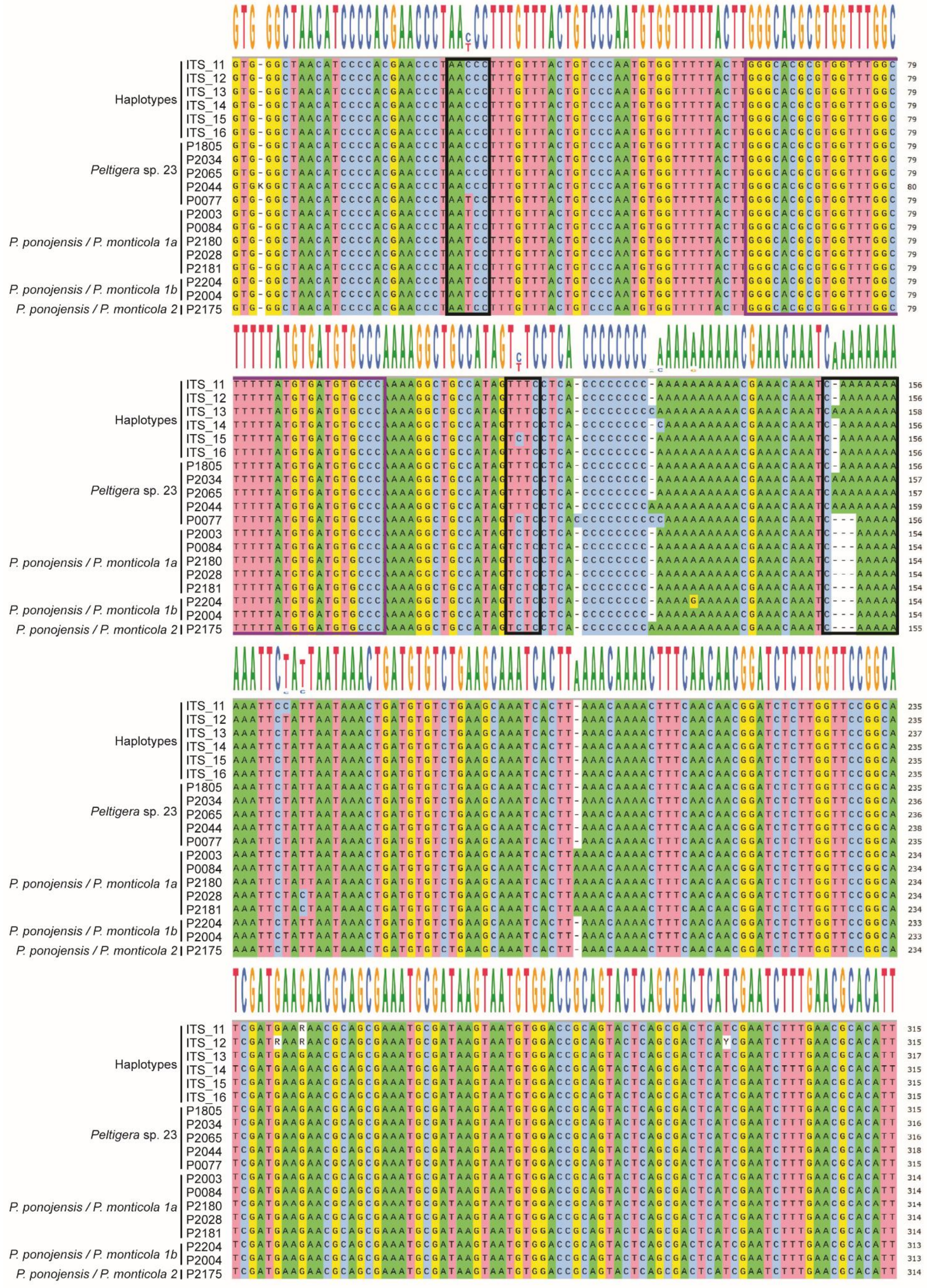
Haplotypes
ITS_11
ITS_12
ITS_13
ITS_14
ITS_15
ITS_16
Peltigera sp. 23
P1805
P2034
P2065
P2044
P0077
P. ponojensis / P. monticola 1a
P2003
P0084
P2180
P2028
P2181
P. ponojensis / P. monticola 1b
P2204
P2004
P. ponojensis / P. monticola 2
P2175

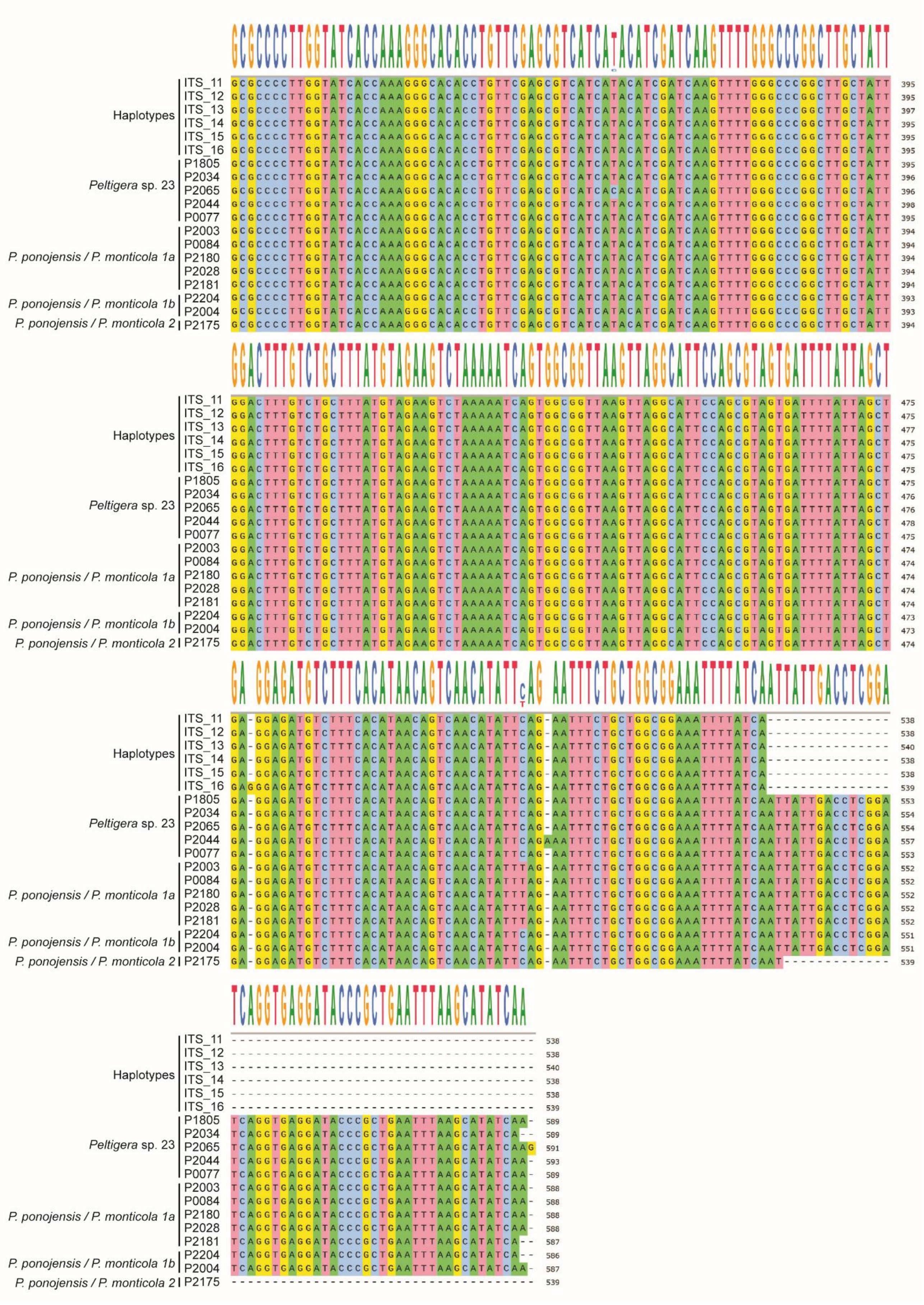

Figure S2. Alignment of ITS haplotypes associated with clade 5 with the closest reference sequences according to phylogenetic analysis. Representatives of *P. antarctica* and *P. ponojensis* / *P. monticola* 1a, 1b and 2 are included.
