# Supplementary figures and images for: High Andean Steppes of Southern Chile Contain Little-Explored Peltigera Lichen Symbionts
Source: J Fungi (Basel). 2023 Mar 18;9(3):372. doi: 10.3390/jof9030372 (PMC10058012; doi:10.3390/jof9030372)

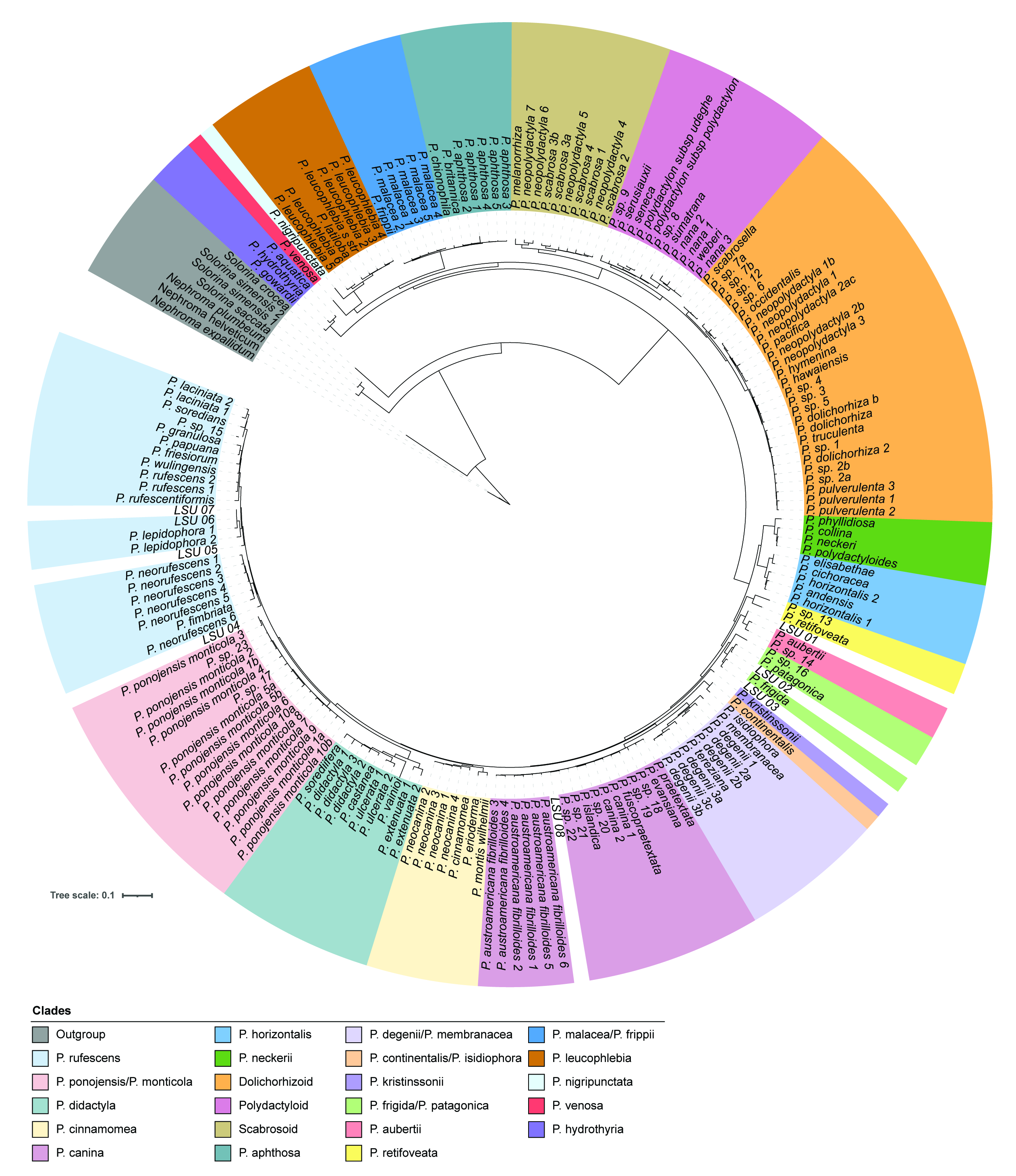

Supplement: Supplementary file 1 [file jof-09-00372-s001.zip › Figure S1.tif]
